# Inequalities in unpaid carer's health, employment status and social isolation

**DOI:** 10.1111/hsc.14104

**Published:** 2022-11-12

**Authors:** Nicola Brimblecombe, Javiera Cartagena Farias

**Affiliations:** ^1^ Care Policy and Evaluation Centre (CPEC) London School of Economics and Political Science (LSE) London UK

**Keywords:** employment and earnings, health, inequalities, social isolation, unpaid/informal care

## Abstract

Providing higher‐intensity unpaid care (higher care hours or care within the household) is associated with negative impacts on people's paid employment, mental health and well‐being. The evidence of effects on physical health is mixed and carer's social and financial outcomes have been under‐researched. The biggest evidence gap, however, is on how outcomes vary by factors other than type or level of care provision, in particular socio‐demographic factors. Our study used two waves of data (2017/19 and 2018/2020) from the United Kingdom Household Longitudinal Study for people aged 16 and older. We investigated the effects of providing care for 10 or more hours a week or within the household in interaction with people's socio‐demographic characteristics. Outcomes included mental and physical health, social isolation, employment status and earnings. We found that caring responsibilities interacted with gender, ethnicity, socio‐economic status (as measured by highest educational qualification), or age to affect carers differentially in a number of areas of their lives leading to, and exacerbating, key disadvantages and inequalities.

**What Is Known about this Topic**

- Providing unpaid care at higher hours or within the household impacts negatively on paid employment, mental health and well‐being
- Findings on association with physical health are mixed
- There is evidence of gender differences for some outcomes but not much is known about inequalities in carer's experiences

**What the Paper Adds**

- Social determinants ‐ identifying as female, ethnic minority or lower socio‐economic group ‐ interacts with care provision to result in greater negative effects on key life domains
- Younger age in conjunction with care responsibilities is associated with poorer mental health and greater social isolation; older age with worse physical health
- Higher‐intensity caring is also associated with higher odds of social isolation or loneliness

## 1 INTRODUCTION

How care needs are met, and disabled and older people enabled to live independent lives, is an important societal and social justice issue. It has had varied traction in the political and policy sphere, although has been higher on the agenda and public consciousness in recent years in many countries, the UK included (Her Majesty's Government, 2014; Scottish Government, 2014; Welsh Government, 2014). Currently, unpaid care comprises the majority of care provided and received (Verbeek‐Oudijk et al., 2014). Discussions, and policy, about the optimal balance between formal and informal care varies across country, time and ideology. However, a consistent part of the equation is the impact on unpaid carers. There is now a substantial body of evidence showing that at higher care hours and/or for co‐resident carers, providing care has significant negative impacts on carer's paid employment, mental health and well‐being (Brimblecombe et al., 2018; Kaschowitz & Brandt, 2017). There are also impacts on carer's physical health when caring for higher hours and/or providing co‐resident care (e.g. Bauer & Sousa‐Poza, 2015), although findings are mixed (Bom et al., 2019) and there is much less longitudinal evidence available. Social and financial outcomes have been much less researched (Spiers et al., 2021). The biggest evidence gap, however, is on how the experiences of carers vary by factors other than type or level of care provision, in particular socio‐demographic factors (Young et al., 2020). The exception is gender where a number of studies have shown that female carers experience greater negative impacts than male carers on their employment (Carmichael & Charles, 2003; Heitmueller & Inglis, 2007; King & Pickard, 2013; van Houtven et al., 2013), mental health (Bauer & Sousa‐Poza, 2015; Bom et al., 2019) and physical health (Bom et al., 2019).

Few of these studies consider care hours but those that do suggest that gender differences in impacts on employment are not solely due to female carers providing higher hours of care than men (King & Pickard, 2013). Mental health was shown to be worse among women providing higher hours of care in a recent longitudinal study in Northern Ireland (Doebler et al., 2017). Cross‐sectional studies show similar patterns (e.g. Office for National Statistics, 2013; Verbakel et al., 2017). Research on locus of care and gender suggests some interaction effect with regard to carer's employment (Arber & Ginn, 1995). In terms of other socio‐demographic characteristics, evidence from a cross‐sectional Swiss study in a very specific context (carers of partners with spinal cord injury) suggested that socio‐economic position was associated with greater perceived strain. This was not moderated by care hours (Tough et al., 2019). A European study using longitudinal data found that carers with higher wealth experienced greater life satisfaction (Brandt et al., 2021) although, in contrast a cross‐sectional study in Japan found no interaction effect of care provision and income on depressive symptoms (Saito et al., 2018). However, in general and in the UK context, the evidence on factors other than gender – for example, age, ethnicity and socio‐economic status (SES) ‐ is scant (Spiers et al., 2021).

Carers are not a homogenous group and the gap in evidence on variations in outcomes matters because of the body of work showing that factors such as age, SES, gender and ethnicity are key determinants of outcomes in many domains (Dahlgren & Whitehead, 1991; Marmot et al., 2020; Solar & Irwin, 2010). In addition, in order to best support the most vulnerable carers, we need to first identify them. Our study investigated the interaction between provision of care and key socio‐demographic factors. In doing so, we utilise a social determinants conceptual framework (Solar & Irwin, 2010). In this framework, socio‐economic position, which comprises social class and social stratifiers (e.g. age, gender, ethnicity, education), is a key structural determinant of outcomes. We also draw on Pearlin and colleagues' stress process model (Pearlin et al., 1990). This model postulates that impacts of care provision depend on both elements of the care itself (e.g. care hours, care type) but also on the context, including socio‐economic position (gender, ethnicity, age, educational attainment) and resources. We know that providing care has impacts on people's lives in several domains and that gender, SES, ethnicity and age also impact people's outcomes. Our study aimed to add to the evidence base by exploring the effects of care provision and socio‐demographic factors in combination to better understand who is most disadvantaged and how experiences differ. We focused on carers providing the most intense care (higher care hours or co‐resident carers) because of evidence showing greater, or sometimes only any, impacts at these levels and types of care provision (Brimblecombe et al., 2018).

## 2 METHODS

**FIGURE 1:**
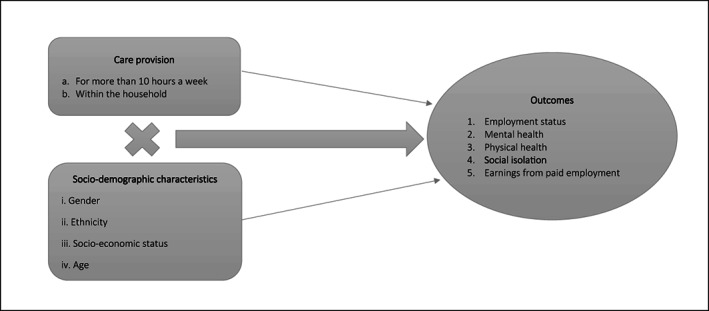
Analysis framework

Our methods strategy was as follows. Using data from the UK Household Longitudinal Study (UKHLS), we identified people aged 16 and older providing unpaid care at time 1 (wave 9; 2017/19) of (a) ten or more hours a week; (b) within the household. We then looked at how interaction of care provision and socio‐demographic at time 1 was associated with a number of outcomes at time 2 (wave 10; 2018/20) (Figure 1).

### 2.1 Data and sample

We used data from Waves 9 (2017/19) and 10 (2018/20) of the UKHLS (University of Essex Institute for Social and Economic Research, 2021). The UKHLS started in 2009 and collects data annually from a sample of household members aged 10 or older living in the UK. Sampling is based on a proportionately stratified, clustered sample of addresses selected by postcode, supplemented by specific additional samples added at subsequent waves (Knies, 2017). Our sample comprised all panel members who took part in the study in both Wave 9 and Wave 10, who were aged 16 or older in Wave 9, and for whom data about caring responsibilities, hours and type were available. The resultant sample for carers caring for 10 or more hours a week was 25,935: 23,586 non‐carers; 2349 carers and for co‐resident carers *N* = 25,354: 23,586 non‐carers; 1768 carers. Attrition is an issue for any longitudinal data collection and whilst UKHLS takes a number of measures to minimise this, there is a degree of attrition between waves. However, whilst here is modest under‐representation among the youngest age groups, men, ethnic minority respondents and those on the lowest incomes, the sample is still considered representative of the UK population (Lynn & Borkowska, 2018).

### 2.2 Measures

#### 2.2.1 Caring responsibilities

The variables for caring responsibilities were derived from three questions asked of respondents at time 1:

- ‘Is there anyone living with you who is sick, disabled or elderly whom you look after or give special help to (for example, a sick, disabled or elderly relative/husband/wife/friend etc.)?’
- ‘Do you provide some regular service or help for any sick, disabled or elderly person not living with you?’

And the follow‐up question:

iii ‘Thinking about everyone who you look after or provide help for, both those living with you and not living with you ‐ in total, how many hours do you spend each week looking after or helping them?’

Non‐carers were defined as people who answered no to both the first two questions. Higher care hour carers were defined as people who answered yes to either or both questions (i) and (ii) and indicated that they were providing care for 10 or more hours a week in question. Co‐resident carers were defined as people who answered yes question (i) (either on its own or together with question (ii)).

Caring for ten or more hours a week was selected because previous research shows that this is the threshold at which impacts on carers are greatest; few or no impacts are observed at lower hours (Brimblecombe et al., 2018; King & Pickard, 2013). In addition, preliminary analysis on the sample showed that providing care at 10 or more hours was the threshold at which negative outcomes in all domains were seen in our data. This effect was seen at the lower range (10–19 h). Within the 10 or more hours category, some higher care hour bands were associated with worse outcomes but this was not linear and no clear pattern emerged. Co‐resident caring was selected because co‐resident care provision is associated with greater impacts on carers than extra‐resident (e.g. Bauer & Sousa‐Poza, 2015; Kaschowitz & Brandt, 2017; Michaud et al., 2010; Nguyen & Connelly, 2014).

### 2.3 Carer characteristics

The carer characteristics used as predictors in the models were gender (male = 0; female = 1); ethnicity (White = 0; Asian = 1; Black = 2); highest educational qualification (degree or higher degree = 0; primary, secondary or no formal qualifications = 1) as a measure of socio‐economic status; and age group (16–24; 25–44; 45–65; 66–74; 75+). Highest educational qualification is a well‐used measure of socio‐economic status and was used in our study because it has a good response rate, is easy to measure and includes people who are unemployed. The social class variables available in UKHLS use information from current job. As a result, over 40% of responses are ‘inapplicable’.

### 2.4 Outcomes

We considered five outcomes separately at time 2: employment status; annual earnings from paid employment; physical health; mental health; and social participation (loneliness or isolation). Employment status was recoded into two categories: 0 = in paid‐ or self‐employment; 1 = not in paid employment. The continuous variable for earnings was based on a question about monthly earnings from paid employment and thus excluded earnings from self‐employment. We multiplied the monthly figure by 12 to give annual earnings. For employment and earnings, we excluded sample members who were in full‐ or part‐time education or training at the same time as paid employment and sample members who were over the UK state pension age at the time (66 and older). For health outcomes, the variables used were the Physical and Mental Components of the Short‐Form 12 Health Survey (SF12, PCS and MCS) which measure physical and mental health, respectively; they have been validated for use in the general population (Ware et al., 1996). Lower scores indicate poorer physical/mental health. Social participation was derived from two questions asked at Wave 10 about how often the person felt lonely or felt isolated from others, recoded into 0 for hardly ever or never and 1 for some of the time or often.

### 2.5 Analysis

First, we used descriptive statistics to report the characteristics of the sample. We then investigated (a) the effect of care provision; (b) the effect of people's socio‐demographic characteristics; and (c) the combined, interactive, effect. We used two‐step multivariate regression models which used the factors on their own and with interaction terms. We looked at care provision and carer characteristics at time 1 (Wave 9) and outcomes at time 2 (Wave 10). Multivariate logistic regression was used for categorical outcome variables: employment status and social participation. Multivariate linear regression was used for continuous outcome variables: physical health score (PCS) and mental health score (MCS). For analysis of earnings, as this variable has a skewed distribution with a substantial number of zeros, we used two‐part Generalised Linear Models (Mullahy, 1998). We used a modified Park test (Manning & Mullahy, 2001) to select the appropriate distribution and link function. The marginal effect of providing care at time 1 on each cost measure at time 2 could then be estimated from each regression model: this represents the mean cost at time 2 associated with a person providing care (of 10 or more hours a week/within the household) at time 1 compared to someone not providing care at time 1.

Models controlled for covariates potentially associated with each outcome, based on previous research where available (e.g. Marmot & Bell, 2012; Pickard et al., 2018) and/or initial bivariate analysis. Covariates varied by outcome but included the carer characteristics listed above excluding the one used in the interaction term as the predictor in each model. In addition, for employment outcomes (employment status, earnings), we included carer's partnership status (single or in a partnership) and health (presence or absence of a ‘long‐standing physical or mental impairment, illness or disability;’ LLTI). In analyses of health outcomes, additional covariates were partnership status and housing tenure (coded as owner‐occupied, social‐rented or privately rented). Social‐rented housing (or ‘public housing’) in the UK is provided at more affordable rents, usually by local government or non‐profit sector housing associations. We used the unweighted sample for the regression analysis. Where sampling weights are solely related to the independent variables, as they are in our models, unweighted estimates are preferred (Winship & Radbill, 1994).

All tests of statistical significance used robust standard errors. A significance level of 0.05 was used as the criterion to determine statistical significance and 0.10 to determine marginal significance. We conducted analyses using Stata 14.2 (StataCorp, 2015).

## 3 RESULTS

Table 1 shows that 66% of carers caring for 10 or more hours week were female and 34% were male. Women also made up a higher proportion of co‐resident carers than men. The majority of higher care hour carers and co‐resident carers identified as White. This was 85% for higher care hours, similar to the proportion in the sample overall (84%) and slightly lower (81%) for co‐resident caring. Asian carers comprised 11% of higher care hour carers and 15% of co‐resident carers. Two‐thirds of carers had primary, secondary or no qualifications, higher than their proportion in the sample overall (57%). Proportions of both higher care hours carers and co‐resident carers were highest in mid‐life (45–64).

**TABLE 1:** Sample descriptives

	Non‐carer and 10+ hours carer sample		Non‐carer and co‐res carer sample	
	*N* = 25,935		*N* = 26,227	
	Non‐carer *N* = 23,586 (90.9%)		Non‐carer *N* = 23,586 (89.9%)	
	Carers for 10+ hours a week *N* = 2349 (9.1%)		Co‐resident carers *N* = 2641 (10.1%)	
	N	%	N	%
Carer characteristics time 1				
Female	1540	65.6	1511	57.2
Male	809	34.4	1130	42.8
White	1946	85.0	2059	80.9
Asian	260	11.4	391	15.4
Black	84	3.7	95	3.7
<degree	1325	65.8	1496	65.7
Degree +	688	34.2	780	34.3
Aged 16–24	86	3.7	229	8.7
Aged 25–44	519	22.1	649	24.6
45–65	1099	46.8	979	37.1
66–74	390	16.6	432	16.4
75+	255	10.9	352	13.3

Table 2 shows the association between providing care for ten or more hours a week and each outcome; the association between outcomes and people's socio‐demographic characteristics; and the combined effect of care provision and each characteristic. As expected, compared to non‐carers, caring for ten or more hours a week were associated with lower odds of being in paid employment; lower earnings; poorer mental and physical health; and higher odds of loneliness and social isolation. However, as the interaction findings show, this effect was amplified in combination with gender, ethnicity, SES or age. The combination of being a carer and being female was, in the main, associated with worse outcomes: poorer mental health; higher odds of feeling lonely or isolated; and lower earnings. Both gender and care provision appeared to contribute to these outcomes. For example, women had lower earnings than men, and carers had lower earnings than non‐carers. The combined effect of being a carer and being female was over £10,000 a year reduced earnings. Similarly, there was a combined carer and ethnicity effect on health and earnings for Asian carers. For Black carers, the picture was more complex. Being Black and providing care was associated with the highest odds of not being in paid employment. However, in our study people from Black ethnic backgrounds had significantly better mental health scores than people from White ethnic backgrounds and the interaction effect of care provision and Black ethnicity on mental health was not statistically significant.

**TABLE 2:** Higher care hour carer's outcomes: Regression models

	Not in paid employment at time 2^1^	Mental health score time 2	Physical health score time 2	Lonely or isolated	Annual net earnings from employment time 2^1^
	Odds ratio (95% CI)	Coefficient (95% CI)	Coefficient (95% CI)	Odds ratio (95% CI)	Mean cost difference (£) (95% CI)
Care responsibilities					
Providing care for 10 or more hours a week compared to non‐carer	**1.45** * 1.30, 1.61	**−1.37** * −1.66, −1.09	**−0.96** * −1.26, −0.66	**1.15** * (1.09, 1.21)	**−4635.04** * −5373.71, −3896.38
Socio‐demographic characteristics					
Male (ref)	—	—	—	—	—
Female	1.0 ns 0.87, 1.15	**−2.08** * −2.37, −1.79	**−0.73** * −1.00, −0.66	**1.35** * (1.28, 1.43)	**−6977.32** * −7408.30, −6546.34
White (ref)	—	—	—		—
Asian	**2.64** * 2.19, 31.8	0.43 ns −0.11, 0.96	**−2.74** * −3.19, −2.30		**−4682.89** * −5288.65, −4077.13
Black	1.23 ns 0.88, 1.71	**2.72** * 1.82, 3.61	0.22 ns −0.54, 0.98		327.22 ns −841.45, 1495.89
Degree or higher educational qualification (ref)	—	—	—		—
Primary, secondary, or no formal qualifications	**3.34** * 2.88, 3.87	−0.21 ns −0.51, 0.08	**−2.32** * −2.59, −2.04		**−7782.90** * −8206.87, −7358.94
Aged 16–24		**−5.29** * −6.14, −4.44	**14.13** * 13.28, 14.98	**2.65** * (2.25, 3.11)	
Aged 25–44		**−5.40** * −6.02, −4.79	**10.74** * 10.00, 11.47	**3.00** * (2.61,3.45)	
Aged 45–65		−**2.94** * −3.53, −2.35	**6.91** * 6.18, 7.64	**1.94** * (1.70,2.23)	
Aged 66–74		0.15 ns −0.48, 0.78	**3.20** * 2.39, 4.02	**1.04 ns** (0.89, 1.21)	
Aged 75+ (ref)	—	—	—	—	
Interaction of care provision and socio‐demographic characteristics					
Male#non‐carer (ref)	—	—	—	—	—
Male#carer	**2.46** * 1.73, 3.51	**−2.02** * −2.93, −1.11	**−1.71** * −2.68, −0.75	**1.18** * 1.01, 1.39	**−6812.39** * −7850.68, − 5774.11
Female#carer	**1.98** * 1.50, 2.60	**−5.13** * −5.13, −4.42	**−2.74** * −3.48, −2.00	**1.87** * 1.65, 2.11	**−10938.84** * −11261.61, −10616.12
White#non‐carer (ref)	—	—	—		—
White#carer	**2.08** * 1.64, 2.63	**−2.72** * −3.32, −2.12	**−2.08** * −2.73, −1.43		**−4988.23** * −5508.89, −4467.58
Asian#carer	**3.71** * 2.04, 6.73	**−2.68** * −4.60, −0.75	**−3.13** * −4.81, −1.45		**−7518.6** * −9165.08, −5872.06
Black#carer	**5.07** * 1.90, 13.55	0.51 ns −2.86, 3.88	−0.34 ns −3.23, 2.54		−1931.4 ns −7551.52, 3688.62
Degree or higher#non‐carer (ref)	—	—	—	—	—
Degree#carer	**2.46** * 1.67, 3.61	**−3.19** * −4.10, −2.27	**−1.79** * −2.73, −0.84		**−5506.15** * −6459.77, −4552.53
Lower qualifications#carer	**6.44** * 4.90, 8.46	**−2.79** * −3.51, −2.07	**−4.86** * −5.11, −3.61		**−12021** * −12382.2, −11659.61
Aged 75 + #non‐carer (ref)		—	—	—	
Aged 16‐24#carer		**−8.61** * −12.52, −4.71	**10.22** * **7.09, 13.35**	**3.22** * 1.99, 5.20	
Aged 25‐44#carer		**−8.52** * −9.88, −7.16	**8.20** * 6.84, 9.57	**5.06** * 3.99, 6.42	
Aged 45‐65#carer		**−5.11** * −6.08, −4.14	**4.48** * 3.37, 5.59	**2.62** * 2.16, 3.18	
Aged 66‐74#carer		**−1.92** * −3.19, −0.65	**2.60** * 1.04, 4.16	**1.67** * 1.26, 2.19	
Aged 75 + #carer		**1.44** ** −3.05, 0.17	−0.29 ns −2.26, 1.67	**1.60** * 1.15, 2.23	

*Note*: Controlling for carer's sex, ethnicity, health (LLTI), marital status, highest qualification, age at time 1 in analysis of employment status and analysis of earnings from paid employment; controlling for carer's sex, ethnicity, marital status, highest qualification, housing tenure at time 1 in analysis of physical health score and mental health score; controlling for sex, health (LLTI), marital status, highest qualification in analysis of social isolation. Physical health score is Physical Component of the Short‐Form 12 Health Survey (SF12 PCS; range 4.62–75.48); lower score = worse physical health. Mental health score is Mental Component of the Short‐Form 12 Health Survey (SF12 MCS; range 0–75.38); lower score = worse mental health. (1) Under State Pension Age only.

Abbreviation: ns, non‐significant.

* *p* < 0.05

** *p* = 0.10.

Lower‐qualified carers were a particularly disadvantaged group (Table 2). The combination of lower qualifications and being a carer for ten or more hours a week resulted in being six times less likely to be in paid employment, an earnings penalty of £12,000 a year, and having significantly lower mental and physical health scores, indicating worse health. The intersection of age and care provision had differential effects on health. The combination of being younger – aged 16–24 or 25–44 – and being a carer was associated with significantly worse mental health, whereas being older and a carer was associated with significantly worse physical health. Being younger and a carer in combination was also associated with higher odds of being lonely or isolated. The interaction of being a co‐resident carer and carer's gender, ethnicity, qualification and age showed similar results to the interaction of providing higher care hours and those characteristics (Table 3). This includes for experiencing loneliness and social isolation.

**TABLE 3:** Co‐resident carer's outcomes: Regression models

	Not in paid employment at time 2^1^	Mental health score time 2	Physical health score time 2	Lonely or isolated	Annual net earnings from employment time 2^1^
	Odds ratio (95% CI)	Coefficient (95% CI)	Coefficient (95% CI)	Odds ratio (95% CI)	Mean cost difference (£) (95% CI)
Care responsibilities					
Providing co‐resident care compared to non‐carer	**1.35** * 1.21, 1.49	**−1.41** * −1.69, −1.14	**−1.19** * −1.48, −0.91	**1.20** * 1.14, 1.26	**−3844.92** * −4582.2 ‐3107.64
Socio‐demographic characteristics					
Male (ref)	—	—	—	—	—
Female	1.03 ns 0.90, 1.18	**−2.09** * −2.38, −1.80	**−0.81** * −1.08, −0.54	**1.35** * 1.28, 1.43	**−7303.23** * −7735.00, −6871.22
White (ref)	—	—	—		—
Asian	**2.48** * 2.06, 2.98	0.40 ns −0.12, 0.92	**−2.66** * −3.10, −2.23		**−4410.12** * −5018.98, −3801.27
Black	1.08 ns 0.77, 1.53	**2.69** * 1.80, 3.58	0.12 ns −0.64, 0.88		401.79 ns −768.66, 1572.24
Degree or higher educational qualification (ref)	—	—	—		—
Primary, secondary, or no formal qualifications	**3.30** * 2.89, 3.76	**−0.26** ** −0.56, 0.03	**−2.34** * −2.62, −2.06		**−7910.16** * −8336.75, −7383.61
Aged 16–24		**−5.30** * −6.14, −4.47	**14.26** * 14.42, 15.10	**2.69** * 2.29, 3.16	
Aged 25–44		**−5.30** * −5.91, −4.69	**10.80** * 10.08, 11.53	**3.03** * 2.64, 3.48	
Aged 45–65		**−2.90** * −3.48, −2.73	**6.95** * 6.23, 7.67	**2.01** * 1.76, 2.30	
Aged 66–74		0.10 ns −0.52, 0.72	**3.22** * 2.41, 4.02	1.06 ns 0.91, 1.24	
Aged 75+ (ref)		—	—	—	
Interaction of care provision and socio‐demographic characteristics					
Male#non‐carer (ref)	—	—	—	—	—
Male#carer	**1.85** * 1.34, 2.54	**−2.27** * −3.06, −1.47	**−1.78** * −2.59, −0.97	**1.32** * 1.15, 1.53	**−3224.32** * −4125.03, −2323.61
Female#carer	**1.84** * 1.39, 2.44	**−5.26** * −5.99, −4.53	**−3.56** * −4.34, −2.78	**2.04** * 1.79, 2.31	**−11125.1** * −11495.78, −10754.39
White#non‐carer (ref)	—	—	—		—
White#carer	**2.08** * 1.65, 2.63	**−2.80** * −3.39, −2.21	**−2.69** * −3.33, −2.05		**−4687.49** * −5218.19, −4156.79
Asian#carer	**2.40** * **1.44, 4.01**	**−2.65** * −4.17, −1.12	**−3.20** * −4.50, −1.90		**−5050.22** * −6424.6, −3675.82
Black#carer	1.62 ns 0.45, 5.87	0.97 ns −2.25, 4.19	−1.64 ns −4.46, 1.18		−1211.48 ns −6560.77, 4137.82
Degree or higher#non‐carer (ref)	—	—	—	—	—
Degree#carer	**1.64** * 1.22, 2.19	**−3.06** * −3.74, −2.32	**−2.23** * −3.11, −1.36		**−4006.08** * −4990.02, −3022.14
Lower qualifications#carer	**6.57** * 4.86, 8.87	**−3.03** * −3.74, −2.32	**−4.90** * −5.64, −4.16		**−11661.91** * −12007.48, −11316.33
Aged 75 + #non‐carer (ref)		—	—	—	
Aged 16‐24#carer		**−8.31** * −10.72, −5.89	**12.64** * 10.81, 14.47	**3.26** * 2.32, 4.56	
Aged 25‐44#carer		**−7.70** * −8.94, −6.46	**8.13** * 6.88, 9.39	**4.70** * 3.77, 5.87	
Aged 45‐65#carer		**−5.40** * −6.40, −4.40	**3.87** * 2.69, 5.05	**3.15** * 2.58, 3.85	
Aged 66‐74#carer		**−3.07** * −4.35, −1.79	**1.86** * 0.28, 3.44	**1.76** * 1.35, 2.30	
Aged 75 + #carer		**−2.29** * −3.66, −0.92	−1.40 ns −3.17, 0.38	**1.49** * 1.11, 2.00	

*Note*: Controlling for carer's sex, ethnicity, health (LLTI), marital status, highest qualification, age at time 1 in analysis of employment status and analysis of earnings from paid employment; controlling for carer's sex, ethnicity, marital status, highest qualification, housing tenure at time 1 in analysis of physical health score and mental health score; controlling for sex, health (LLTI), marital status, highest qualification in analysis of social isolation. Physical health score is Physical Component of the Short‐Form 12 Health Survey (SF12 PCS; range 4.62–75.48); lower score = worse physical health. Mental health score is Mental Component of the Short‐Form 12 Health Survey (SF12 MCS; range 0–75.38); lower score = worse mental health. (1) Under State Pension Age only.

Abbreviation: ns, non‐significant.

* *p* < 0.05

** *p* = 0.10.

## 4 DISCUSSION

The effects of providing unpaid care at higher care hours or within the household on carer's employment, earnings and mental health are well‐established in the literature and our study findings concur. However, our study adds to that body of evidence by showing that gender, ethnicity, SES and age interact with care provision to amplify these effects. That is that care and context contribute to outcomes (as postulated by Pearlin's stress process model (Pearlin et al., 1990)) and that social determinants also influence carer's outcomes. A further contribution of our paper is the finding that care provision at higher hours or within the household was associated with poorer physical health or greater social isolation; outcomes which are much less studied. Interaction effects were seen here as well leading to greater impacts for some population sub‐groups.

The interaction of being female and providing higher hours of care or providing care within the household was associated with impacts in all life domains under study with the exception of employment status. Previous research shows a greater negative impact for female carers on employment (Carmichael & Charles, 2003; Heitmueller & Inglis, 2007; King & Pickard, 2013; van Houtven et al., 2013), although only the King and Pickard study takes account of care intensity. Our findings of an earnings penalty for female carers are consistent with the few other studies, most not in the UK context, which found differential earnings effects by gender (Keating et al., 2014). The effect of higher hours of care provision and co‐resident caring on earnings are likely due to the fact that these types of care are associated with a lower likelihood of being in paid employment and a higher likelihood of working reduced hours and/or in lower‐paid jobs (Brimblecombe et al., 2018; Keating et al., 2014). This is exacerbated for female carers by the gender pay gap whereby women earn on average 15% less than men in the UK (Office for National Statistics, 2020a). The amplifying effect of the interaction may be due to female carers being more likely to reduce their paid employment hours and/or to take a less well‐paid job (Keating et al., 2014). There is evidence that strategies with regards to managing employment and care differ by gender with men more likely to organise care round their work and women their work around care (Auth et al., 2019).

The types of care provided also may vary by gender. Women provided higher hours of care in our study and may also provide more personal care. Whilst this is part of the caring context per se, it is also related to the unequal gendered nature of caring and to gender roles and is therefore currently inextricably, but not irrevocably, linked with gender. We also observed an interaction effect of being female and providing care on mental and physical health and on social isolation. The care effect is likely to be due to the mental and physical stresses and strains of providing high‐level or within‐household care and reduced time to spend on social participation. The interaction effect may be due to gender roles, differential access to resources (Solar & Irwin, 2010), and to the complex relationship between gender roles and coping strategies, agency and gender differences in self‐care (Zygouri et al., 2021). A recent review found that female carers found it harder to maintain a sense of self‐agency and ‘*felt socially restricted in pursuing their interests, personal needs and career ambitions’* (Zygouri et al., 2021). As with employment, differences in the type of care may also play a part, in particular women providing more emotional care as well as higher hours.

For ethnicity, the picture was more complex. Being Asian and providing higher hours or within‐household care had negative effects on health and on earnings. The effect on earnings may be due to a combination of the negative effect of caring on employment and on earnings, the ethnic pay gap and different strategies and pressure with regards to the balance of care and work. The effect on health is consistent with ethnicity being a social determinant of health (Marmot & Bell, 2012; Solar & Irwin, 2010) and the effect of care provision on health. Ethnicity was not associated with differences in care hours provided but there may be other differences in type of care provision for Asian carers that may contribute to the outcomes seen. The findings for mental health and Black ethnicity were unexpected. Despite a wealth of existing evidence on ethnicity and health leading us to expect poorer outcomes for Black participants compared to White (Bignall et al., 2019), the findings for Black carers and mental health score were non‐significant in our study. Black participants in the UKHLS sample overall had higher mental health scores than White participants, indicating better mental health. This may be due to methodological issues. There is higher attrition among minority ethnic participants, for example, although attrition in the sample is unrelated to health status (Lynn & Borkowska, 2018), and a higher proportion of missingness for the mental health variable Black participants in our sample were younger on average than White participants and a higher proportion were female. However, neither of these help explain our finding because women, and younger people, had poorer mental health in our study. Black participants also had higher educational qualifications, which is associated with better mental health.

SES, as measured by the highest educational qualification, was on its own and in intersection with care provision associated with negative impacts in every domain. Lower qualifications are associated with lower earnings and employment rates (Office for National Statistics, 2020b) and are a major determinant of health (Marmot & Bell, 2012; Solar & Irwin, 2010). The interaction effect of care provision and qualification on employment and earnings may also be because higher qualified carers are less likely to have flexible work practices or be able to negotiate them (Spiess & Schneider, 2003). A further possible reason for the interaction effect of care provision and SES on mental and physical health may be the role of choice. Theories of, and evidence on, role captivity, role strain and choice suggest that reduced choice about taking on care responsibilities negatively impacts on carer's well‐being (Al‐Janabi et al., 2018). Lower qualified people may have less resources available to them and therefore less alternatives to providing that care themselves. The stress process model also sets out how the resources available to carers can increase or decrease the impacts of caring on well‐being (Pearlin et al., 1990). The choices available to lower qualified carers are not just due to their lower financial and other resources. Choice is also delineated by cultural and familial expectations and these may vary by SES. Expectations about who provides care also vary by ethnicity and gender (della Giusta et al., 2009; Parveen et al., 2011). Greater role captivity may thus also be a contributor to the interaction effect for female and Asian carers both because of the pressure of societal expectations and because women and ethnic minorities are less likely to seek or receive care services and thus to have fewer alternatives to providing that care themselves (Greenwood et al., 2014; Zygouri et al., 2021).

The effects of age and the interplay between age and care provision were striking. The combination of age and caring responsibilities mean that younger carers had much worse mental health than older carers whereas older carers had poorer physical health. The interaction effect may be linked to a combination of younger people's and carer's poorer mental in general. However, it may also result in part from care provision among younger carers being particularly linked to lack of alternatives (Olsen, 2000) and from their fewer emotional, financial and other resources to mediate the effects of providing care (Aldridge, 2018). Carers aged 25–44 had similarly poorer mental health. This may also be due to role strain and need to juggle competing commitments of work and childcare; such factors exacerbate the stresses of care provision (Brimblecombe et al., 2018; Pearlin et al., 1990). That caring exacerbates mental ill health among younger carers and physical ill health among older carers is a cause for concern and for action. In our study, the odds of younger people expressing being lonely was higher than older people and the combined effect of care and age was seen most in younger age groups for both higher care hour carers and co‐resident carers. Care provision at higher hours will reduce the time available for social participation. Stigma and fear of being judged, particularly among younger carers, may cause concerns about bringing people home and/or disconnection from their non‐carer peers (Becker & Becker, 2008; Joseph et al., 2020).

Thus caring on its own was, in our study, associated with poorer outcomes 1 year later, both in domains where a wealth of evidence has already shown this (employment and mental health) and in domains where there has been a research gap (physical health; social isolation). However, some carers were doubly disadvantaged. Structural disadvantage, role captivity, choice, alternatives and opportunities and financial and other resources may help to explain why. The SDH framework shows how social determinants contribute to health outcomes in several key ways. Some authors have argued that because of the extensive effects of care provision on health and well‐being, unpaid caring should also be considered a social determinant, as part of living and working conditions (Spiers et al., 2021).

Our study has some limitations. Whilst a strength is that we look at the interaction between care responsibilities and individual social determinants of health, because of sample sizes and the difficulties of interpretation we were unable to look at further interaction between, for example caring, gender and ethnicity. Clearly, there will be variation within these broad sub‐groups and further research would benefit from exploring some of these intersections, as also pointed out by Hengelaar et al in their 2021 review of intersectionality and unpaid care. Even within the broad sub‐groups used, there are small sample sizes for some groups, another limitation. There are a number of other limitations to our study. The earnings variable only includes earnings from paid employment, thereby excluding people in self‐employment (4.8% of the sample). We sought to address possible selection bias in a number of ways – our regression models considered care provision at time 1 and outcomes at time 2, controlling for a number of factors suggested from previous research as likely to be associated with providing care and with the outcomes under study (e.g. Marmot & Bell, 2012; Pickard et al., 2018). Cost estimates were based on two‐part models which have been shown to be robust to endogenous selection (e.g. Drukker, 2017). However, there is still the potential for some selection bias. The analytical tools that are commonly used for addressing any further bias were not suitable for this analysis. For example, fixed effects models would not have enabled us to consider the effect of characteristics that tend to remain the same in both waves e.g. ethnicity and gender. Propensity score matching has disadvantages for this study because it only allows for analysis of carers new to caring at time 1. Care hours are much lower in a sample matched like this, a disadvantage when exploring higher‐intensity caring. This, plus the shorter duration of care provision, makes the matched sub‐sample substantially different from carers more generally as well as reducing the sample size. Furthermore, we were not seeking to investigate causality, rather how care provision and people's socio‐demographic characteristics interacted to affect outcomes, how experiences differ and who experienced poorer outcomes. Our study has strengths, in particular, that it is based on the analysis of longitudinal data for a large nationally representative sample. This means, for example, that we had sufficient data on provision of care at one time‐point, socio‐demographic characteristics and a wide range of consequences 1 year later.

## 5 CONCLUSION

In conclusion, we found that caring responsibilities interact with socio‐demographic factors to affect carers differentially in a number of life domains leading to, and exacerbating, key disadvantages and inequalities. Our findings reinforce the need for differentiation of carer support. One clear example is the need for mental health support and prevention for younger carers and physical health support and prevention for older carers. One of the pathways by which social factors determine health and other outcomes is by the ability to access health, long‐term care and other services (Solar & Irwin, 2010) and there is evidence of differential access to care services among carers and the people they support (Floridi et al., 2021; García‐Gómez et al., 2015; Ilinca et al., 2017). Thus a further implication is a need to reduce or remove barriers to support for sub‐groups of carers, examples being through targeting and/or changes to charging regimes and other barriers. Because caring responsibilities are a contributory factor to poorer outcomes, good and targeted support for carers including services for the person they care for has an important role to play. However, support for carers is just one part of what is needed. For example, for female or ethnic minority carers, the gender or ethnic pay gap may be as much of an issue as their care responsibilities. Future qualitative research might fruitfully explore in depth some of the reasons for the sub‐group differences seen in our study.

## CONFLICT OF INTEREST

The authors declare no conflicts of interest.

## ETHICS

Ethical approval for the UKHLS data used in this study was obtained by the University of Essex Ethics Committee which has approved all data collection on the UKHLS main study and innovation panel waves.

## Data Availability

The data that support the findings of this study are available from the UK Data Service. Restrictions apply to the availability of these data, which were used under licence for this study. Data are available from the UK Data Service and only be accessed with the permission of the UK Data Service.

## References

[hsc14104-bib-0001] Aldridge, J. (2018). Where are we now? Twenty‐five years of research, policy and practice on young carers. Critical Social Policy, 38(1), 155–165. 10.1177/0261018317724525

[hsc14104-bib-0002] Al‐Janabi, H. , Carmichael, F. , & Oyebode, J. (2018). Informal care: Choice or constraint? Scandinavian Journal of Caring Sciences, 32(1), 157–167. 10.1111/scs.12441 28401583 PMC5873411

[hsc14104-bib-0003] Arber, S. , & Ginn, J. (1995). Gender differences in the relationship between paid employment and informal care. Work, Employment and Society, 9(3), 445–471. 10.1177/095001709593002

[hsc14104-bib-0004] Auth, D. , Leiber, S. , & Leitner, S. (2019). “The interrelation of class, ethnicity, gender, and employment in coping with care: An intersectional analysis addressing family caregivers in Germany,” in *4th Transforming Care Conference 24–26 June 2019*. Copenhagen, Denmark.

[hsc14104-bib-0005] Bauer, J. M. , & Sousa‐Poza, A. (2015). Impacts of informal caregiving on caregiver employment, health, and family, IZA discussion paper 8851. Forschungsinstitut zur Zukunft der Arbeit Institute for the Study of Labor.

[hsc14104-bib-0006] Becker, F. , & Becker, S. (2008). Young adult carers in the UK: Experiences, needs and services for carers aged 16–24. Princess Royal Trust for Carers.

[hsc14104-bib-0007] Bignall, T. , Jeraj, S. , Helsby, E. , & Butt, J. (2019). Racial disparities in mental health: Literature and evidence review. Race Equality Foundation and Health and Well‐being Alliance. https://raceequalityfoundation.org.uk/wp‐content/uploads/2022/10/mental‐health‐report‐v5‐2.pdf

[hsc14104-bib-0008] Bom, J. , Bakx, P. , Schut, F. , & van Doorslaer, E. (2019). The impact of informal caregiving for older adults on the health of various types of caregivers: A systematic review. The Gerontologist, 59(5), e629–e642. 10.1093/geront/gny137 30395200 PMC6850889

[hsc14104-bib-0009] Brandt, M. , Kaschowitz, J. , & Quashie, N. T. (2021). Socioeconomic inequalities in the wellbeing of informal caregivers across Europe. Aging & Mental Health, 26, 1–8. 10.1080/13607863.2021.1926425 34010061

[hsc14104-bib-0010] Brimblecombe, N. , Fernández, J. , Knapp, M. , Rehill, A. , & Wittenberg, R. (2018). Review of the international evidence on support for unpaid carers. Journal of Long‐term Care, 25–40. https://journal.ilpnetwork.org/articles/3/galley/3/download/

[hsc14104-bib-0011] Carmichael, F. , & Charles, S. (2003). The opportunity costs of informal care: Does gender matter? Journal of Health Economics, 22(5), 781–803. 10.1016/S0167-6296(03)00044-4 12946459

[hsc14104-bib-0012] Dahlgren, G. , & Whitehead, M. (1991). Policies and strategies to promote social equity in health. Institute for Future Studies.

[hsc14104-bib-0013] della Giusta, M. , Hashimzade, N. , & Jewell, S. (2009). Why care? Social norms and the supply of unpaid care. Department of Economics, University of Reading.

[hsc14104-bib-0014] Doebler, S. , Ryan, A. , Shortall, S. , & Maguire, A. (2017). Informal care‐giving and mental ill‐health ‐ differential relationships by workload, gender, age and area‐remoteness in a UK region. Health & Social Care in the Community, 25(3), 987–999. 10.1111/hsc.12395 27753162

[hsc14104-bib-0015] Drukker, D. M. (2017). Two‐part models are robust to endogenous selection. Economics Letters, 152, 71–72. Accessed: August 29, 2019. https://www.sciencedirect.com/science/article/pii/S0165176517300046

[hsc14104-bib-0016] Floridi, G. , Carrino, L. , & Glaser, K. (2021). Socioeconomic inequalities in home‐care use across regional long‐term Care Systems in Europe. Journals of Gerontology: Series B, 76(1), 121–132. 10.1093/geronb/gbaa139 PMC7756692 32996570

[hsc14104-bib-0017] García‐Gómez, P. , Hernández‐Quevedo, C. , Jiménez‐Rubio, D. , & Oliva‐Moreno, J. (2015). Inequity in long‐term care use and unmet need: Two sides of the same coin. Journal of Health Economics, 39, 147–158. 10.1016/j.jhealeco.2014.11.004 25544399

[hsc14104-bib-0018] Greenwood, N. , Habibi, R. , Smith, R. , & Manthorpe, J. (2014). Barriers to access and minority ethnic carers' satisfaction with social care services in the community: A systematic review of qualitative and quantitative literature. Health and Social Care in the Community, 23, 64–78. 10.1111/hsc.12116 25135207 PMC4283974

[hsc14104-bib-0019] Heitmueller, A. , & Inglis, K. (2007). The earnings of informal carers: Wage differentials and opportunity costs. Journal of Health Economics, 26(4), 821–841. 10.1016/j.jhealeco.2006.12.009 17276532

[hsc14104-bib-0020] Hengelaar, A. H. , Wittenberg, Y. , Kwekkeboom, R. , van Hartingsveldt, M. , & Verdonk, P. (2021). Intersectionality in informal care research: A scoping review. Scandinavian Journal of Public Health, 14034948211027816. 10.1177/14034948211027816 PMC9903248 34232094

[hsc14104-bib-0021] Her Majesty's Government . (2014). Care Act 2014. The Stationery Office. http://www.legislation.gov.uk/ukpga/2014/23/contents/enacted/data.htm

[hsc14104-bib-0022] Ilinca, S. , Rodrigues, R. , & Schmidt, A. E. (2017). Fairness and eligibility to long‐term care: An analysis of the factors driving inequality and inequity in the use of home Care for Older Europeans. International Journal of Environmental Research and Public Health, 14(10), 1224. 10.3390/ijerph14101224 29036885 PMC5664725

[hsc14104-bib-0023] Joseph, S. , Sempik, J. , Leu, A. , & Becker, S. (2020). Young Carers research, practice and policy: An overview and critical perspective on possible future directions. Adolescent Research Review, 5(1), 77–89. 10.1007/s40894-019-00119-9

[hsc14104-bib-0024] Kaschowitz, J. , & Brandt, M. (2017). Health effects of informal caregiving across Europe: A longitudinal approach. Social Science & Medicine, 173, 72–80. 10.1016/j.socscimed.2016.11.036 27930918

[hsc14104-bib-0025] Keating, N. C. , Fast, J. E. , Lero, D. S. , Lucas, S. J. , & Eales, J. (2014). A taxonomy of the economic costs of family care to adults. Journal of the Economics of Ageing, 3, 11–20. 10.1016/j.jeoa.2014.03.002

[hsc14104-bib-0026] King, D. , & Pickard, L. (2013). When is a carer's employment at risk? Longitudinal analysis of unpaid care and employment in midlife in England. Health and Social Care in the Community, 21(3), 303–314. 10.1111/hsc.12018 23356685

[hsc14104-bib-0027] Knies, G. (2017). Understanding society. The UK household longitudinal study: Waves 1–7. User guide. Institute for Social and Economic Research, University of Essex.

[hsc14104-bib-0028] Lynn, P. , & Borkowska, M. (2018). Some indicators of sample representativeness and attrition bias for BHPS and understanding society. Institute for Social and Economic Research, University of Essex.

[hsc14104-bib-0029] Manning, W. G. , & Mullahy, J. (2001). Estimating log models: To transform or not to transform? Journal of Health Economics, 20(4), 461–494. 10.1016/s0167-6296(01)00086-8 11469231

[hsc14104-bib-0030] Marmot, M. , Allen, J. , Boyce, T. , Goldblatt, P. , & Morrison, J. (2020). Health equity in England: The Marmot review 10 years on. Institute of Health Equity.

[hsc14104-bib-0031] Marmot, M. , & Bell, R. (2012). Fair society, healthy lives. Public Health, 126, S4–S10. 22784581 10.1016/j.puhe.2012.05.014

[hsc14104-bib-0032] Michaud, P. C. , Heitmueller, A. , & Nazarov, Z. (2010). A dynamic analysis of informal care and employment in England. Labour Economics, 17(3), 455–465. 10.1016/j.labeco.2010.01.001

[hsc14104-bib-0033] Mullahy, J. (1998). Much ado about two: Reconsidering retransformation and the two‐part model in health econometrics. Journal of Health Economics, 17(3), 247–281. 10.1016/s0167-6296(98)00030-7 10180918

[hsc14104-bib-0034] Nguyen, H.T. and Connelly, L.B. (2014) “The effect of unpaid caregiving intensity on labour force participation: Results from a multinomial endogenous treatment model,” Social Science & Medicine. 2014/01/22, 100, 115–122. 10.1016/j.socscimed.2013.10.031. 24444846

[hsc14104-bib-0035] Office for National Statistics . (2013). The gender gap in unpaid care provision: Is there an impact on health and economic position? Office for National Statistics. http://www.ons.gov.uk/peoplepopulationandcommunity/healthandsocialcare/healthandwellbeing/articles/fullstorythegendergapinunpaidcareprovisionisthereanimpactonhealthandeconomicposition/2013‐05‐16

[hsc14104-bib-0036] Office for National Statistics . (2020a). Gender pay gap in the UK: 2020. Office for National Statistics. https://www.ons.gov.uk/employmentandlabourmarket/peopleinwork/earningsandworkinghours/bulletins/genderpaygapintheuk/2020

[hsc14104-bib-0037] Office for National Statistics . (2020b). Labour force survey. Office for National Statistics.

[hsc14104-bib-0038] Olsen, R. (2000). Families under the microscope: Parallels between the Young Carers debate of the 1990 s and the transformation of childhood in the late nineteenth century. Children and Society, 14, 384–394.

[hsc14104-bib-0039] Parveen, S. , Morrison, V. , & Robinson, C. A. (2011). Ethnic variations in the caregiver role: A qualitative study. Journal of Health Psychology, 16(6), 862–872. 10.1177/1359105310392416 21415259

[hsc14104-bib-0040] Pearlin, L. I. , Mullan, J. T. , Semple, S. J. , & Skaff, M. M. (1990). Caregiving and the stress process: An overview of concepts and their measures. Gerontologist, 30(5), 583–594. 10.1093/geront/30.5.583 2276631

[hsc14104-bib-0041] Pickard, L. , Brimblecombe, N. , King, D. , & Knapp, M. (2018). ‘Replacement Care’ for Working Carers? A Longitudinal Study in England, 2013–15. Social Policy and Administration, 52(3), 690–709. 10.1111/spol.12345

[hsc14104-bib-0042] Saito, T. , Kondo, N. , Shiba, K. , Murata, C. , & Kondo, K. (2018). Income‐based inequalities in caregiving time and depressive symptoms among older family caregivers under the Japanese long‐term care insurance system: A cross‐sectional analysis. PLoS One, 13(3), e0194919. 10.1371/journal.pone.0194919 29590211 PMC5874058

[hsc14104-bib-0043] Scottish Government . (2014). Carers (Scotland) Act. Scottish Government.

[hsc14104-bib-0044] Solar, O. , & Irwin, A. (2010). A conceptual framework for action on the social determinants of health, social determinants of health discussion paper 2 (policy and practice). World Health Organization. http://apps.who.int/iris/bitstream/10665/44489/1/9789241500852_eng.pdf?ua=1&ua=1

[hsc14104-bib-0045] Spiers, G. , Liddle, J. , Stow, D. , Welsh, O. , Kunonga, P. , Beyer, F. , Craig, D. , Ramsay, S. , & Hanratty, B. (2021). Caring as a social determinant of health. Findings from a rapid review of reviews and analysis of the GP patient survey. Public Health England.

[hsc14104-bib-0046] Spiess, K. , & Schneider, U. (2003). Interactions between care‐giving and paid work hours among European midlife women, 1994 to 1996. Ageing and Society, 23(1), 41–68. 10.1017/S0144686X02001010

[hsc14104-bib-0047] StataCorp . (2015). Stata statistical software: Release 14. StataCorp LP.

[hsc14104-bib-0048] Tough, H. , Brinkhof, M. W. G. , Siegrist, J. , Fekete, C. , & Group, for the S.S . (2019). Social inequalities in the burden of care: A dyadic analysis in the caregiving partners of persons with a physical disability. International Journal for Equity in Health, 19(1), 3. 10.1186/s12939-019-1112-1 31892324 PMC6938621

[hsc14104-bib-0049] University of Essex Institute for Social and Economic Research (2021) Understanding Society: Waves 1‐10, 2009‐2019 and Harmonised BHPS: Waves 1‐18, 1991‐2009. SN: 6614. UK Data Service. 10.5255/UKDA-SN-6614-14

[hsc14104-bib-0050] van Houtven, C. H. , Coe, N. B. , & Skira, M. M. (2013). The effect of informal care on work and wages. Journal of Health Economics, 32(1), 240–252. 10.1016/j.jhealeco.2012.10.006 23220459

[hsc14104-bib-0051] Verbakel, E. , Tamlagsrønning, S. , Winstone, L. , Fjær, E. L. , & Eikemo, T. A. (2017). Informal care in Europe: Findings from the European social survey (2014) special module on the social determinants of health. European Journal of Public Health, 27(suppl_1), 90–95. 10.1093/eurpub/ckw229 28355645

[hsc14104-bib-0052] Verbeek‐Oudijk, D. , Woittiez, I. , Eggink, E. and Putman, L. (2014) Who cares in Europe? A comparison of long‐term care for the over‐50 s in sixteen European countries. The Geneva Association.

[hsc14104-bib-0053] Ware, J. J. , Kosinski, M. , & Keller, S. D. (1996). A 12‐item short‐form health survey: Construction of scales and preliminary tests of reliability and validity. Medical Care, 34(3), 220–233. 10.1097/00005650-199603000-00003 8628042

[hsc14104-bib-0054] Welsh Government . (2014). Social Services and Well‐being (Wales) Act 2014. Welsh Government.

[hsc14104-bib-0055] Winship, C. , & Radbill, L. (1994). Sampling weights and regression analysis. Sociological Methods & Research, 23(2), 230–257. 10.1177/0049124194023002004

[hsc14104-bib-0056] Young, H. M. , Bell, J. F. , Whitney, R. L. , Ridberg, R. A. , Reed, S. C. , Vitaliano, P. P. , & Hepburn, K. (2020). Social determinants of health: Underreported heterogeneity in systematic reviews of caregiver interventions. Gerontologist, 60, S14–S28. 10.1093/geront/gnz148 32057083 PMC7019663

[hsc14104-bib-0057] Zygouri, I. , Cowdell, F. , Ploumis, A. , Gouva, M. , & Mantzoukas, S. (2021). Gendered experiences of providing informal care for older people: A systematic review and thematic synthesis. BMC Health Services Research, 21(1), 730. 10.1186/s12913-021-06736-2 34301248 PMC8306003

